# Repertory of the Back

**Published:** 1898-04

**Authors:** E. H. Wilsey

**Affiliations:** Parkersburg, W. Va.


### REPERTORY OF THE BACK.

E. H. Wilsey, M. D., Parkersburg, W. Va.

(Continued from page 132.)

**Tired,** aching feeling and some burning in back and legs in women, *Pic-ac.*

— feeling in lower part of back, Onos.

— dull tired ache in shoulders and across back, *Curare.*

— aching across small of back and limbs; knees ache, *Hydrast.*

— bruised feeling in muscles of back extending from point of scapula to ilium on each side of spine, better by firm pressure, Vib-op.

— feeling in small of back, *Pallad.;* when walking, Camph.

— arms and back tired, and sore all over, Sarrac.

— pain in small of back, *Sepia.*

— feeling in small of back with pain, and on sitting down he had to sit very straight, Sep.

— wandering pain in muscles of back, worse on left side, Vib-op.

— feeling in back, impeding motion, Magnolia.

— feeling in back, *Nat-m.*

— pain and tired feeling in small of back as if bruised, *Sul.*

**Toe,** small pimples on back and third toe with sore pain when touched, Zinc.

**Tongs,** twisting and griping in small of back as from tongs, then pain in arms and feet as if they would turn outward, Graph.

**Tolerate,** can hardly tolerate any heat near back, *Phos.*

**Touched,** soreness in small of back when touched, *Colchi.*

— during cough sensation as if lungs touched back, Sul.

**Torn,** sensation as if vertebræ were being torn apart when stooping forward and then when bending backward again when walking, *Chel.*

**Touch,** violent tearing pain in back and limbs, worse from least touch, *Chel.*

**Transverse,** lancination in small of back in transverse direction, Cup.

— sharp stitches transversely through small of back close above hip, Nat-m.

**Treading,** drawing pain in back on moving and treading, Sul-ac.

**Trembling** in back during fever, *Eup-per.*

— in back, *Coccul.*, Lil-tig.

— with warmth of back and between shoulders, Cof-cr.

**Tumor,** a peculiar tumor in central part of back growing on a pedicle as large as a cherry, bluish in color, ½ an inch long, *Con.*

**Turn,** bruised pain at night toward morning in back, dare not turn over, Nat-c.

— has to sit up to turn in bed on account of backache, *Nux-v.*

— so lame in back in region of liver it is almost impossible to turn in bed; better by motion, Diosc.

— pain in small of back, cannot turn over in bed at night or stoop without pain, Zinc.

**Twinging** in back or any part of body, *Zinc.*

**Twinges** up back; better by drawing the shoulders backward; worse by reverse, Cycla.

— near dorsal vertebra, *Amyl-nit.*

**Twist,** pains at the waist as if she would break in two; run up whole left side of body like lightning, seems as if they would give her a twist while running up, with pain in elbow and wrist, *Cornus.*

**Twisting,** and griping in small of back as from tongs, then pain in arms and feet as if they would turn outward, Graph.

**Twitches,** violent pains along whole back, stitches and twitches so she could not stoop nor pick up anything with her hand; increased by inspiration, Alum.

**Twitching,** sharp stitch in left side of back and at times in left thigh, Stann.
— a violent tearing and twitching in the nape proceeding gradually down the back and then ceasing, Mag-c.
— in back when asleep, Amm-c.
— in small of back, Ratan.
— in muscles of back, Xanthox.
— stinging, piercing in back and sacrum, taking away breath, Caust.
— sensation in back and nape toward the head, Nat-m.
— violent cramps and convulsive twitching of the arm and back, which scarcely intermit for a moment, Iod.

**Ulcers,** scrofulous on back, *Cist.*

**Undressing,** vesicles on back with intense itching exciting to scratch, especially in evening on undressing, Nat-c., Nat-s.

**Uneasiness,** great and stiffness in small of back, Petrol.

**Urging** in back, *Calc-c.*
— or burning in anus with aching in small of back, Merc-per.

**Urinate,** drawing extending from back to hips with urging to urinate, *Lach.*

**Urine,** unable to hold urine after being struck on back by falling from wagon, Hyper.

**Urination,** relieves backache, *Lyco.*
— backache during urination, *Ant-cr., Kali-b., Ipec.*
— backache with slight incontinence of urination, *Thuya.*

**Uterine,** weakness when walking from uterine trouble, *Sepia.*
— pain in back with uterine hemorrhage, Trill.

**Uterus,** pains in back with heaviness in uterus, Aloe.
— pain in uterus with pain in small of back very violent, with pressure as in labor, Inula.
— pain in back between hips and a slight numb feeling in uterus at 6 P. M., Calabar.
— reflex spinal irritation from uterus, *Phos.*

**Uterus,** pain in lower part of back, piercing drawing through to uterus, *Helon.*

**Vaccination,** backache after, *Sil.*

**Variola**, aching in back with variola, *Ant-t.*, *Variol*, Ant-cr.

**Varix,** strong pressure in back before the protrusion of a varix from the rectum, Alum.

**Vertebra,** pain in right side of seventh vertebra, Ver-vir.

**Vesicles** on back with intense itching exciting to scratch, especially in evening on undressing, Nat-c.

**Violent** stitches in back, Hepar.

— pains in top of right shoulder and shortly afterward in back from about first to fourth dorsal vertebra, *Lob-i.*

— pain in back from nape to sacrum, Chel.

— pain in small of back, *Sil.*

— pain in back by day but worse at night, so that she can only lie on her side; worse by speaking and by breathing deeply, Nat-c.

— pain in back and especially in sacral region, Sec.

— pain in head from back down spine, Rhus-t.

**Vice**, small of back very painful, feels as if in a vice, not allowing him to lie still at night or in daytime, has to sit in bent position, *Kali-iod.*

— sensation as if small of back was in vice, Am-m., Æthu.

**Vomit,** he awakens at 2 A. M. with a febrile rigor and hot dry skin, and at times a shivering ague down back from the nape and over the chest, then some sleep from which he awakens in a gentle perspiration, with a pressive pain in back, and hips and in abdomen with inclination to vomit, Hepar.

**Waking** with a severe pain in lower part of back; it is often five minutes before she can straighten, Lac-can.

— stiffness in back at night on waking, Sali-ac.

— aching on waking, better by getting up with disinclination to move, Lappa.

**Walk,** does not walk erect, stoops or bends forward, *Sul.*

— weak child does not learn to walk, All-sat.

— oppression and anxiety when she walks somewhat quickly, with perspiration on back and chest, Nit-ac.

— perspires very profusely, especially on his back, when he walks or exercises otherwise, Nat-c.

**Walking** almost impossible, causing backache, *Æscul.*

— weakness when walking with uterine trouble, *Sepia.*

— inner coldness in small of back and loins; worse by walking but a few steps, *Camph.*

— pain particularly with stiffness; better by walking, *Sepia.*

— tired feeling in small of back when walking, Camph.

— debility and weakness in back and lower limbs; better by walking, *Hydr.*

— stiffness in back after horseback ride, stooping or walking, and lumbar region, Lyc.

— burning pressure in back; worse when walking in open air, Kali-c.

— obliged to stoop when walking, and gets painful stitches in back, by accidentally kicking against something, *Sepia.*

— back pains as if broken after walking an hour, Plat.

— in small of back, stitches and pain as from a sprain during rest and ceasing on walking, Staph.

— pain in back after walking, Phos.

— lancinating pain in back while walking, Cof-cr., Sul.

— pain in small of back; worse by motion and walking, Mag-c.

— pain in small of back when walking so she could not walk straight, Am-m.

— violent pain in small of back, when walking was frequently obliged to stand still, though it was steadily better by continuing to walk, *Zinc.*

— gets out of bed at 4 A. M. on account of pain in back; better after rising and walking about, *Nux-v.*

**Walking,** unbearable pains in back recurring periodically and hindering in walking, Phos.

— pain in back while sitting; better by walking or lying, *Cobalt.*

— pressure and burning in back; better by walking; worse by sitting and lying in bed, Nitrum.

— cracking in small of back while walking, Zinc.

— stiffness of back on sitting a long time; better by walking, Sul.

— weakness in back and loins when walking, *Graph.*

— lower part of back lame from walking or standing, Zing.

— stitch like jerk in small of back when walking, extends toward hips rather than upper part; worse by sitting or standing, *Fer.*

— pressive stitches in back; worse while walking or stooping and more on rising, Rhus-t.

— back and knees feel bruised while lying still in bed; better on rising and walking about, *Puls.*

— burning in back while walking in open air when she gets warm, Sil,

— a feeling at noon while walking as if a weight were lying across shoulders weighing him down so that his head sank forward, Carb-sul.

— heat on cheeks and flushes of heat on back in evening while walking in open air, Phos-ac.

— backache while walking, feels as if she must give up and lie down, *Kali-c.*

— violent backache after walking, *Nat-c.*

— sensation as if vertebræ were being torn apart when walking, *Chel.*

— backache; worse when stooping or walking, and from drinking coffee, *Cham.*

— difficulty in walking, increasing until child cannot walk, *Phos.*

— stiffness of back; better by walking, Copaiba.

**Walking,** pain in back when walking like from pressure when sitting or stooping, Borax.

— pain in small of back while walking, Alum.

— pain in small of back; worse from walking or standing, *Kali-c.*

**Waist,** severe pains across back and between shoulders and waist at 2 P. M., Lyss.

**Warm,** pain in back, loins and lower part of thigh in evening when entering a warm room, Gels.

— sensation passed up back into head, Sarrac.

**Warmth,** a feeling of warmth in lower part of back and small of back, as if lumbar region was asleep, extending down in sacrum, hips, and post. portion of thighs, *Berb.*

— with trembling of back and between shoulders, Cof-cr.

**Warts,** dreams that his back is covered with warts and excrescences, Mez.

**Water,** sensation as if hot water was creeping through back from below upward, Nitr-sp-d.

— chilly along back like cold water, Stram.

— sensation as if cold water was dropping down back, *Cap.*

— as if cold water was poured down back with headache, Alum.

— as if cold water were poured down back, *Puls.*, Zinc.

— as if cold water were spurted on back at 6 P. M., *Lyco.*

**Weak,** pain in small of back as if lame and weak, *Lach.*

— back feels too weak to support body, Ox-ac.

— back feels weak as after taking a long journey, Chin-ars.

— back and headache in morning, Pallad.

— back feels weak as if it would soon give out, *Phos.*

— back very weak, unable to stand erect, Physos.

— stiff aching back, Med.

— feeling in back, Tell.

— wants to lean on something, back weak, Sarrac.

— feeling in small of back; worse from mental annoyances, *Cal-c.*

**Weak,** child does not learn to walk, back is weak, All-s.

**Weakness** of back undefined, Crotal., All-s., Æscul-g., Chrom-ac., Sarrac., Petrol., Zinc., Sul-ac., Sil., Iris-v., Ver-v., Pod., Zing., Con., Diosc., Abrot., Med., Carbo-v., Tell., Plant., Chin-ars., Pallad., Pop-c., Cimex-lec., Apis., Ole-jec., Sep., Calab., Physos., Nat-ph., Ox-ac., *Murex., Pic-ac., Agari., Cal-c., Ham., Hydras., Gels., Graph., Cascar., Eup-per., Lyss., Phos., Arg-n., Kali-c., Puls., Coccul., Nat-m.*

— stiffness of cervical muscles and great weakness of back, *Coccul.*

— of cervical muscles with heaviness of head, *Coccul.*

— and numbness in back, Agari.

— when walking, from uterine trouble, *Sepia.*

— in small of back, *Calc-c.,* Petrol., Eup-perf.

— and debility in back and lower limbs; better by walking about, *Hydrast.*

— in small of back on beginning to walk and transient weakness in lower extremities, Zinc.

— and feeling of tension in small of back while sitting, with tension in head, Zinc.

— in back and limbs with sleepiness, *Gels.*

— in back and loins on walking, *Graph.*

— in back, can hardly stand alone, Sul-ac.

— and backache, inclination to lie down, a distaste for business, *Casc.*

— of back and paralyzed feeling in lower limbs, could scarcely walk, Sil.

— in lower part of back, Iris-v.

— of back, Curare., Ver-v., Carbo-v.

— backache as from weakness; better when leaning against something, Zing.

— in back and sacrum with profuse vaginal discharge when walking and sitting, Graph.

**Weakness** and lameness in small of back and subsequent lassitude, Con.
— in small of back, lame and stiff, Diosc.
— great in back as if it would split and fall apart, *Lyss.*
— and soreness of back, Podo.
— of small of back extending around body as if broken, Plant.
— violent bruised pain in back while walking in open air, Zinc.
— and pain in small of back, Psor., *Sepia.*
— and great pain in small of back at 6 P. M., *Sepia.*
— dull heavy pain, with great weakness in small of back, *Lil-tig.*
— drawing like a painful weakness in small of back, and spine while sitting or stooping, Zinc.
— and pain in back; also down arms at 2 P. M., Calab.
— and paralytic sensation in small of back, Phos.
— great in legs, with backache, especially at night, *Arg-n.*
— great in small of back and lower limbs, *Kali-c.*, Nat-m.
— early on rising, like paralysis in small of back; sometimes also near abdomen, *Nat-m.*

**Weariness** in left side of back in morning on moving left arm, Sul.
— and stiffness of back with seminal emissions; excited sexual desire, *Puls.*
— burning in small of back, Poplus-c.
— and heaviness in back while lying down, Phos.
— sensation of weariness and soreness in small of back, Cimex.
— pain in back and lower limbs as if beaten in morning on rising, with general weakness, Stann.
— while sitting, with peculiar pressing pain in back in region of last rib, Canth.

**Weather,** small of back pains; worse when sitting and in wet weather, *Rhod.*

**Weeping,** violent pain in small of back like gnawing, drawing up between the shoulders, where it becomes so violent she feels like weeping, Alum.

**Weight,** painful tension and stiffness in cervical muscles with a sensation as if weight were lying upon them, Vinca-min.

— stiffness, lameness and pain in back, *Helon.*

— sensation of weight in back and shoulders with dyspnœa, *Nat-m.*

— in back and pelvis on rising from a seat, Aloe.

— as if an iron bar was pressing on small of back, Elaps.

**Wet,** severe rheumatic stitches from getting wet while warm, *Dulc.*

**Wheals,** violent itching on back, lower limbs and nates in evening in bed, with wheals after scratching, which soon pass off, Lyco.

**Wind,** collection of wind pressing against back, *Cup-s.*

**Wood,** pain in small of back as if a piece of wood, lying crosswise, were being pressed out, *Nux-m.*

**Woolen,** sensation on skin of back as if he wore a stout woolen shirt, Fer-sul.

**Work,** exhausted as after hard work, especially in back, Apis.

— backache after hard work, *Arn.*

— cannot work on account of backache, *Asaf.*

**Writing,** pain like stiffness in small of back when writing; better by becoming erect, Laurocer.

**Yawning,** restlessness great with yawning and stretching of arms and legs with pain in back; weariness as from too great exertion, with awkward tottering gait with jerks taking away breath or going into abdomen, *Lach.*

— acids cause pain in back with yawning and stretching as in fever, Lach.

— stiff pain in cervical muscles on moving neck and on yawning, *Coccul.*

**Yawning**, shivering in back in A. M. with frequent yawning and inclination to sleep, Graph.

— after yawning a sensation in small of back as if something elastic like air was pressing to get out, Amm-m.

— cold trickling down on both sides of upper arm, over the back and feet while yawning, Mez.

**Years**, constant backache for several, Sul.

**Zoster**, eruption on back like zoster, *Cist-can.*

